# Normal Blood Flow in Rat Abdominal Aorta: An Ultrasound Study

**DOI:** 10.3390/biomedicines13061385

**Published:** 2025-06-05

**Authors:** Anna A. Dokuchaeva, Kseniya S. Podolskaya, Irina Yu. Zhuravleva

**Affiliations:** Institute of Experimental Biology and Medicine, «E.Meshalkin National Medical Research Center» of the Ministry of Health of the Russian Federation («E.Meshalkin NMRC»), 15, Rechkunovskaya str., Novosibirsk 630055, Russia; podolskaya_k@meshalkin.ru (K.S.P.); juravl_irina@mail.ru (I.Y.Z.)

**Keywords:** hemodynamics, ultrasound examination, blood flow velocity, abdominal aorta, Wistar rats

## Abstract

**Background/Objectives:** Ultrasound Doppler diagnostics is a modern diagnostic method, routinely used to determine the blood flow velocity in vessels in clinical practice and scientific experiments. The aim of this study was to investigate hemodynamics and identify blood flow velocity norms for various age groups of animals in the range of two to six months. **Methods:** This study presents the blood flow velocity characteristics in different areas of the abdominal aorta of 30 growing Wistar rats two to six months old. The data from senile rats aged 24 months were used as a reference, since these animals should not show any changes in hemodynamics associated with the active growth of the organism. Ultrasound screening in the group of growing rats was performed monthly, and blood flow velocity was measured at three points: proximal to the renal arteries, distal to the renal arteries, and before the bifurcation zone of the abdominal aorta. **Conclusions:** The obtained data can be used as normative values in in vivo small-diameter tissue-engineered vascular graft (TEVG) studies to assess changes in hemodynamics.

## 1. Introduction

A Wistar rat is a common and affordable model object for in vivo studies of small-diameter tissue-engineered vascular grafts (TEVGs), investigating such features as sustainability, thrombogenicity, tissue response, and vascular compliance [1]. In rats, the experimental prostheses are usually implanted in the infrarenal abdominal aorta [2,3]. Apparently, the data obtained in this model cannot be directly transferred to humans, but it is appropriate to conduct an initial screen using small mammals before conducting studies in more complex and expensive models close to humans, such as monkeys or minipigs. As a rule, for a comprehensive assessment of the dynamics of artificial vascular matrix remodeling, its biomechanical characteristics, and in vivo performance, ultrasonographic methods are used in combination with a number of methods, including histological, physical, and physicochemical tests [4,5]. The method of ultrasound Dopplerography is common for assessing the hemodynamics and functional sustainability of the graft [6]. It is non-invasive, widely available, easy to perform, and relevant for determining the blood flow characteristics [7,8]. Unlike other methods based on intravascular measurements, it does not require preliminary surgical operations or catheterization of the vessel, which in itself is a stress factor that can distort the results of the study, especially in terms of pathological process differentiation [9]. On the contrary, ultrasound diagnostics can be used repeatedly during the experiment, which allows for dynamic control of hemodynamic parameters without unnecessary invasions [10]. These principles are true for both human and animal studies, which explains the choice of the ultrasonic method for the aims of this research.

Echo-Doppler has great diagnostic value for solving such problems, since it allows researchers to study the flow in a specific area and obtain a detailed analysis of the flow distribution, while simultaneously measuring the blood flow parameters and visualizing them in the form of graphs [11]. In experiments, it is also necessary to take the translational features of animal models into account [12]. The life cycle of rats is as short as 2.5–3 years, and the average life expectancy of a human is 70–80 years. Thus, the ratio of rat lifespan to human age is approximately 1:30. Hence, the age of a six-month-old animal is equivalent to eighteen years for a human, the age of twelve-month-old animals is equal to thirty years, and twenty-four months—to sixty years [13].

In experiments with tissue-engineered vascular grafts (TEVG) on rats, the infrarenal abdominal aorta is the most preferable position for implantation [14]. This allows the avoidance of prolonged renal ischemia when the aorta is clamped during surgery, and, accordingly, the complications associated with it. The proximal anastomosis is applied below the renal arteries, the distal one is located just above the aortic bifurcation. This model uses TEVGs with a diameter of 1.5–2 mm. For ultrasound monitoring of the implant function in experiments lasting more than 3 months, in addition to direct visualization of the graft (qualitative characteristic), the characteristics of blood flow in the aorta are usually assessed at three points: proximally to the proximal anastomosis, at the level of the graft, and distally to the distal anastomosis [3,14]. Increasing the linear flow velocities allows for a quantitative assessment of potential hemodynamic disturbances in the implantation area.

In this regard, it is important to note the presence of age-related norms of the aortic wall state and blood flow characteristics to differentiate normal and pathological parameter values in the graft area. To evaluate the results of the experiment correctly, it is very important to minimize the influence of factors associated with the growth and development of the animal (e.g., aorta/graft diameter mismatch). For this, the age of inclusion of the animal in the experiment should coincide with the age at which all hemodynamic indicators have already stabilized. Despite the fact that ultrasonic research methods in small animals are constantly being improved, specific data on age-dependent norms of blood flow velocity in the abdominal aorta of Wistar rats are not unified and hard to find in the literature [15,16,17].

The aim of this study was to determine the monthly dynamics of blood flow characteristics in the abdominal aortas of intact Wistar rats from two to six months of age, as the animals matured, and to determine the normal values for each age group.

## 2. Materials and Methods

All experimental procedures were carried out in accordance with the EU Directive 2010/63/EU for animal experiments and were approved by the Ethics Committee of E. Meshalkin National Medical Research Center (protocol No 3, 15 June 2021).

### 2.1. Animals

The experimental group consisted of 30 healthy male Wistar rats. The first study was performed at the age of two months, then repeated monthly as the animals grew, up to six months. Ten adult (24 months) intact Wistar rats with stable hemodynamics were studied as a control group. Blood flow measurements in this group were performed in the same way.

The body weight of the examined animals depending on age is given in Table 1.

### 2.2. Ultrasound Diagnostics

The ultrasound examination was performed after combined anesthesia, administered via intramuscular injections of Telazol (Zoetis Manufacturing & Research Spain, S.L., Gerona, Spain) 5 mg/kg and Rometar (SPOFA, Praha, Czech Republic) 20 mg/kg. This allowed high-quality immobilization of animals without side effects that could influence the results of the study.

To provide proper adhesion of the sensor to the skin, the fur in the abdominal area of the animal was shaved with a veterinary trimmer (Moser, Unterkirnach, Germany). The study area was covered with a contact gel for ultrasound, Akugel (OOO MediKraft, Berdsk, Russia). The ultrasound procedures and Doppler measurements were carried out using a Philips CX50 portable vascular ultrasound machine (Philips, Bothell, WA, USA) using a standard technique with a 5.0 cm linear sensor L 12-3, with adjustable frequency (3–12 MHz). The animal was placed on its right side, and the ultrasound examination was performed transabdominally on the left side.

The screening was performed along the entire vessel from the anatomical origin (where it enters the diaphragm) to the bifurcation zone (where it divides into the right and left common iliac arteries). Blood flow velocity was measured at three points of the abdominal aorta: proximal to the renal arteries (proximal), distal to the renal arteries (central), and in front of the bifurcation zone of the abdominal aorta (distal), which made it possible to obtain detailed data on the blood flow velocity throughout the vessel. These points correspond to those used in the actual assessment of graft function; the location of the graft corresponds to the central point of the study. It should be noted that the grafts are usually implanted in the second area, past the renal arteries and before to the bifurcation zone, which explains our choice of examination areas. The dynamics of the results for each rat were recorded individually.

### 2.3. The Studied Ultrasound Parameters

The overall scanning was performed using a linear sensor in B-mode, PW (pulse (spectral) Doppler), and color mode were used to obtain information on the flow direction and its velocity (high and low speed) and visualize turbulent flows. The combination of these techniques provided an overall understanding of the blood flow throughout the entire vessel and allowed us to build its complete color map. To obtain the desired view of the vessel, the control scanning volume, the scanning angle, Doppler beam tilt, the pulse frequency, and spectral inversion were adjusted during the scanning. The obtained graphs were optimized automatically.

Blood flow velocities, expressed in mm/s were measured in PW mode. Hemodynamics in the abdominal aorta were assessed by the following parameters:

PSV—peak systolic velocity—blood flow velocity at the height of systole;

MDV—minimal diastolic velocity—minimal value of blood flow velocity in the diastole phase;

EDV—end diastolic velocity—maximal blood flow velocity at the end of diastole;

TAV—time-averaged blood flow velocity—average velocity of blood particles in the flow, averaged over the cardiac cycle. This parameter can only be measured automatically;

RI—resistance index—the ratio of the difference between the maximum systolic and end diastolic to the maximum systolic blood flow velocity. RI is calculated for each animal as:

$$\mathrm{RI}=\frac{\left(\mathrm{PSV}-\mathrm{EDV}\right)}{\mathrm{PSV}}$$

HR—the mean heart rate.

### 2.4. Statistics

Statistical analysis was performed using the Statistica 13 software (Dell Software Inc., Round Rock, TX, USA). The results were shown as a mean and standard deviation (σ). The non-parametric Mann–Whitney U test was used as a statistical solution with a statistical significance set at *p* < 0.05. The prognostic curves were built in Microsoft Excel 2016 (16.0.5439.1000) (Microsoft, Redmond, WS, USA), with use of the “tendency” option.

## 3. Results

**Figure 1:**
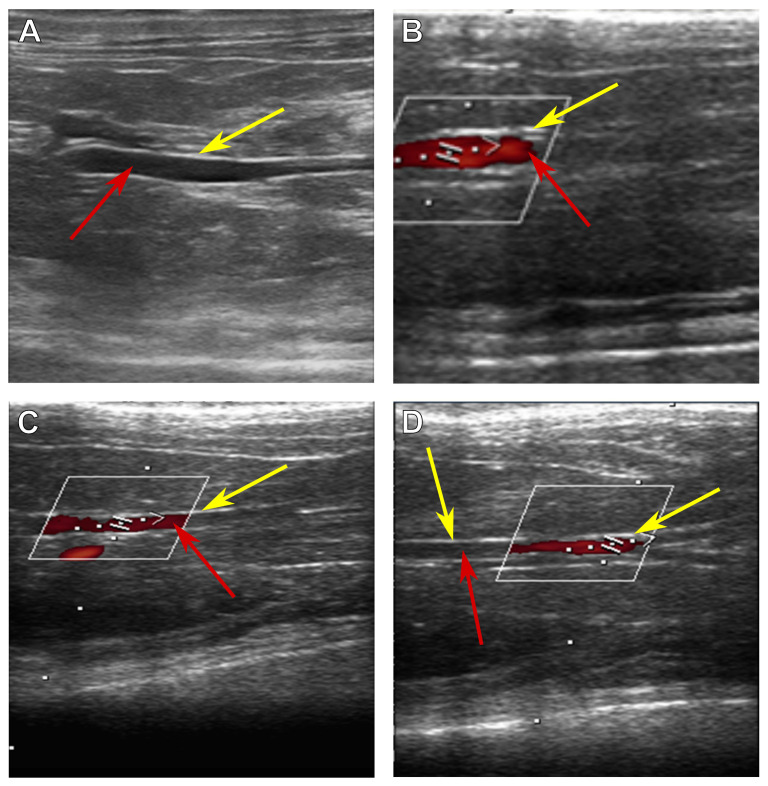
Ultrasonic images of the abdominal aorta of a Wistar rat. (**A**) An overall image, (**B**) proximal to the renal arteries, (**C**) distal to the renal arteries, and (**D**) before the bifurcation zone. Yellow arrows indicate aortic wall, and red arrows indicate the lumen of the vessel. The white frame encloses the blood flow visualization area (red shapes).

The abdominal aorta was visualized as a tubular anechoic formation with an echogenic wall (Figure 1).

In all studied animals, the topography of the aorta in the abdominal cavity corresponded to the standard: the vessel is located along the midline of the vertebral column, dorsal to the caudal vena cava, and is overlapped by the left renal vein. Parietal and visceral peripheral branches extending from the aorta were visualized along the entire length of the vessel. The parietal branches include the caudal diaphragmatic, lumbar, iliolumbar, median caudal, and common iliac arteries. Visceral branches include the celiac, cranial and caudal mesenteric arteries, cranial and caudal adrenal branches, renal, and testicular arteries.

The parameters characterizing the blood flow for growing animals at the ages of two, three, four, five, and six months, and for adult animals at twenty-four months are presented in Table 2.

**Figure 2:**
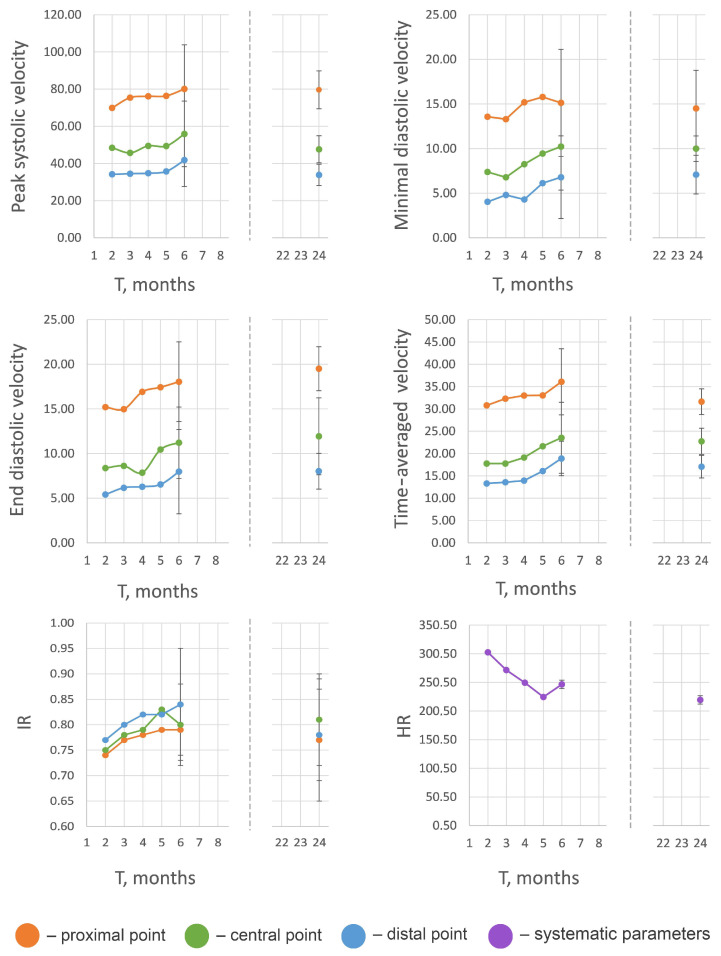
Visualization of age-related changes of blood flow parameters in growing and adult Wistar rats. Separate solid dots represent parameter values, obtained from two-year-old rats. Velocity values (cm/s) and heart rate values (bpm) are given as mean ± σ, cm/sec.

The obtained values of hemodynamic parameters show the tendency for blood flow velocity to increase proportionally to the animal’s age (Table 2, Figure 2), except the heart rate values, as this parameter does not depend on the state of the vessel. HR decreases with aging, which is apparently characteristic of all mammals and is known, in particular, for humans [18,19].

A small gradual increase in blood flow velocities was observed until the 6th month, that is, until the age when the active growth phase ends and the aging process begins [20]; the high individual variability of blood velocity parameters in the groups aged 2–6 months should be mentioned. The results of the reference group (24 months) featured less individual variability and demonstrated no significant difference (*p* > 0.05) in comparison with the group aged 6 months; in some cases, the blood flow velocities and RI were even a little lower than in the 6-month-old animals (Figure 2).

## 4. Discussion

In this study, we present age-associated values of blood flow velocity parameters in juvenile, healthy, and intact Wistar rats aged from two to six months and in adult animals aged two years, in order to compare the hemodynamics between the growing and the adult organism. It was found that during the first six months, there was a steady increase in blood flow velocity in the suprarenal and infrarenal areas and in the distal section of the vessel before the bifurcation of the abdominal aorta, but age-related changes level this tendency in adult animals. The experiments were carried out without stress loads or other intentional effects on hemodynamics, including intravenous anesthesia, so the results can be considered physiological.

In rats aged 2 to 6 months, a gradual increase in all blood flow velocity parameters at all three measurement points was observed (Table 2, Figure 2). We suggest that this effect is due to the cessation of active growth of the organism and the onset of the aging process, which is accompanied by an increase in the rigidity of the aortic walls, an increase in resistance of the vessel wall to blood flow, and a decrease in the elasticity of the wall, which leads to an increase in blood pressure [17,21,22].

The high individual variability is apparently associated with the different growth and maturation rates of the rats. This is also evidenced by the fairly high variability in body weight at 5 and 6 months (Table 1).

At the age of six months, rats reach their active reproductive period, but at this stage growth processes are completed, the aging processes of the body are activated, and changes in the structure of the vessel walls and, accordingly, in hemodynamic parameters, occur. Hemodynamic parameters also stabilize, so the indicators presented in this work correspond to the preoperative examination data published by us earlier [3]. Changes in the blood flow velocity in older animals can be associated with morphological changes in the aortic wall typical for the end of the growth period and the beginning of aging: thickening, mineralization, or fibrosis, which leads to a loss of elasticity and an increase in the coefficient of its resistance to blood flow [23]. Age-related thickening of the arterial walls occurs due to a number of factors, including the dysfunction of complex proteins and of the exchange of macroelements (in particular, calcification) [24]. The rupture of elastin fibers at the level of *t. media* due to numerous factors, including the mechanical effect of constant pulsating wall tension, also has a negative effect on the vessel elasticity. As elastin fibers become impaired, the load transfers to more rigid collagen fibrils, which directly contributes to a significant increase in arterial wall stiffness throughout life [25,26,27,28,29]. The age-related increase in calcium content, resulting in direct binding of calcium ions to elastin fibers, with further deposition of calcium inside the arterial wall, also reduces the extensibility of the vessel [30]. The mechanisms of age-dependent vascular calcification are yet unknown, but it is suggested that inflammation and oxidative stress may play a significant role in this process [31]. Thus, all these factors lead to increased stiffness of the abdominal aortic wall, decreased elasticity, increased vascular resistance to blood flow, and increased blood pressure, which in turn lead to increased blood flow velocity in the vessel [32].

The increase in the abdominal aortic blood flow velocity in rats at the age of six months can be associated with high metabolism, rapid passage through the stages of active growth (an animal at the age of twelve months is considered senile and is usually removed from breeding), and the quick ageing of these animals. Senile changes can explain the decrease in blood flow velocity values for animals at the age of twenty-four months compared with six months as well as the mismatch between the prognostic figures and the empirical results of the study of adult animals [33]. Yet it should be mentioned that, according to the acquired data, no statistically significant difference was found, and, therefore, the hemodynamics in this time period can be considered stable. Commonly, studies on age-related changes in arteries are carried out with animal models; consequently, data concerning humans are scarce and contradictory [34]. However, in the context of current knowledge, it can be inferred that the processes of aging and changes in the vascular wall in different species occur in a similar way [35,36].

In terms of the practical application of the obtained results, we suggest six-month-old rats as optimal for experimental vascular graft implantation. At this age, the diameter of the aorta is surgically appropriate, the aorta blood flow velocities are stable, respectively, and significant changes in the blood flow characteristics during the observation period indicate a pathological process. The lifespan of these rats (2–2.5 years) also provides a long time range for observation. For the most accurate assessment of the results, we recommend performing ultrasound Doppler before graft implantation, immediately after surgery, and then at control time points throughout the mid- or long-term experiment. The dynamics of the results should be recorded individually for each animal.

## 5. Conclusions

The main changes in blood flow velocity in Wistar rats occur at the juvenile age, resulting in a steady increase in all of the studied velocity parameters in the range of 2 to 6 months. The peak values were registered at the age of six months, after which the hemodynamics stabilize.

Five parameters describing the hemodynamics of the abdominal aorta were chosen for this study: peak systolic velocity, end diastolic velocity, minimal diastolic velocity, time-averaged blood flow velocity, and resistance index. These parameters can be used to track blood flow changes in various experiments, investigate in vivo vascular graft implantation, and differentiate occurring pathological processes from the effects of aging. Specific changes in these values can also serve in determining the level and degree of hemodynamic disturbances.

For research involving experimental vascular graft implantation, the animal age group of six months old is recommended.

## Figures and Tables

**Table 1:** Mass to age correlation in experimental groups (grams, mean ± standard deviation).

Age	Body Weight, g ± m
2 months (8 weeks)	126 ± 3.47
3 months (12 weeks)	214 ± 4.48
4 months (16 weeks)	304 ± 4.6
5 months (20 weeks)	430 ± 13.43
6 months (24 weeks)	530 ± 21
24 months (104.29 weeks)	742 ± 8.09

**Table 2:** Linear blood flow velocities of growing and adult Wistar rats, by age; velocity values are given as mean ± σ, cm/s.

Parameter *	Investigation Area	2 Months	3 Months	4 Months	5 Months	6 Months	2 Years
PSV	Proximal	69.88 ± 13.45	75.41 ± 17.33	76.14 ± 15.75	76.37 ± 18.87	80.11 ± 23.66	79.60 ± 10.20
	Central	48.45 ± 10.12	45.71 ± 10.94	49.46 ± 12.48	49.38 ± 11.94	55.89 ± 17.63	47.62 ± 7.24
	Distal	34.16 ± 7.38	34.51 ± 6.58	34.76 ± 8.27	35.72 ± 7.71	41.81 ± 14.24	33.85 ± 5.74
EDV	Proximal	15.21 ± 2.90	14.98 ± 4.78	16.91 ± 3.25	17.43 ± 3.50	18.05 ± 4.46	19.51 ± 2.46
	Central	8.38 ± 3.73	8.62 ± 4.49	7.86 ± 4.71	10.46 ± 4.86	11.22 ± 4.92	11.94 ± 4.31
	Distal	5.41 ± 3.27	6.17 ± 3.50	6.29 ± 6.20	6.54 ± 4.61	7.98 ± 4.72	8.02 ± 2.01
MDV	Proximal	13.57 ± 2.65	13.30 ± 6.09	15.19 ± 4.17	15.78 ± 4.16	15.12 ± 6.57	14.97 ± 4.28
	Central	7.38 ± 3.44	6.78 ± 4.28	8.26 ± 3.86	9.45 ± 4.42	10.23 ± 4.88	9.99 ± 1.42
	Distal	4.03 ± 3.34	4.80 ± 3.48	4.29 ± 3.04	6.13 ± 4.44	6.79 ± 4.63	7.08 ± 2.17
TAV	Proximal	30.80 ± 3.66	32.32 ± 5.66	33.02 ± 5.08	33.05 ± 5.62	36.08 ± 7.41	31.64 ± 2.88
	Central	17.78 ± 4.77	17.78 ± 4.28	19.10 ± 4.01	21.66 ± 5.58	23.54 ± 7.94	22.75 ± 2.93
	Distal	13.31 ± 2.25	13.59 ± 3.88	13.95 ± 2.86	16.07 ± 5.07	18.91 ± 3.86	17.07 ± 2.53
RI	Proximal	0.74 ± 0.05	0.77 ± 0.05	0.78 ± 0.07	0.79 ± 0.08	0.79 ± 0.05	0.77 ± 0.12
	Central	0.75 ± 0.10	0.78 ± 0.13	0.79 ± 0.05	0.83 ± 0.11	0.80 ± 0.08	0.81 ± 0.09
	Distal	0.77 ± 0.12	0.80 ± 0.15	0.82 ± 0.08	0.82 ± 0.11	0.84 ± 0.11	0.78 ± 0.09
HR, bpm		303 ± 7.81	272 ± 5.13	250 ± 23.43	225 ± 5.69	247 ± 5.20	220 ± 5.03

* PSV—peak systolic velocity; MDV—minimal diastolic velocity; EDV—end diastolic velocity, TAV—time-averaged blood flow velocity; RI—resistance index; and HR—heart rate.

## Data Availability

Dataset available on request from the authors.
